# EDIM-TKTL1/Apo10 Blood Test: An Innate Immune System Based Liquid Biopsy for the Early Detection, Characterization and Targeted Treatment of Cancer

**DOI:** 10.3390/ijms18040878

**Published:** 2017-04-20

**Authors:** Johannes F. Coy

**Affiliations:** Zyagnum AG, Lyoner Straße 20, D-60528 Frankfurt am Main, Germany; johannes@dr-coy.de; Tel.: +49-176-4343-0632

**Keywords:** liquid biopsy, phagocytosis, macrophage, EDIM, transketolase, TKTL1, DNase, DNaseX, Warburg, Szent-Györgyi

## Abstract

Epitope detection in monocytes (EDIM) represents a liquid biopsy exploiting the innate immune system. Activated monocytes (macrophages) phagocytose unwanted cells/cell fragments from the whole body including solid tissues. As they return to the blood, macrophages can be used for a non-invasive detection of biomarkers, thereby providing high sensitivity and specificity, because the intracellular presence of biomarkers is due to an innate immune response. Flow cytometry analysis of blood enables the detection of macrophages and phagocytosed intracellular biomarkers. In order to establish a pan-cancer test, biomarkers for two fundamental biophysical mechanisms have been exploited. The DNaseX/Apo10 protein epitope is a characteristic of tumor cells with abnormal apoptosis and proliferation. Transketolase-like 1 (TKTL1) is a marker for an anaerobic glucose metabolism (Warburg effect), which is concomitant with invasive growth/metastasis and resistant to radical and apoptosis inducing therapies. The detection of Apo10 and TKTL1 in blood macrophages allowed a sensitive (95.8%) and specific (97.3%) detection of prostate, breast and oral squamous cell carcinomas. Since TKTL1 represents a drugable target, the EDIM based detection of TKTL1 enables a targeted cancer therapy using the vitamin derivatives oxythiamine or benfo-oxythiamine.

## 1. Introduction

The ability to determine biomarkers in macrophages in the blood using flow cytometry has opened the doors to a completely new form of non-invasive diagnostics (“liquid biopsy”), with which it is possible to detect tumors earlier and characterize their malignancy. Through a “pan-cancer test”, malignancies can be detected through the presence of two biomarkers that indicate fundamental biophysical processes and are changed in all forms of malignancies [1,2]. This allows medical professionals to assess the success of a surgical tumor removal [3] as well as detect recurrences early on [4], so that courses of therapy may be monitored better. Simultaneously, biomarkers allow medical professionals to identify resistance and adapt treatments for each patient. Therefore, an accurate selection of therapies can significantly improve their success.

A successful treatment of tumors is very strongly influenced by the time point of detection of malignancies and the correct choice of treatment. Imaging measures such as radiological examination of the breast (mammography) are applied for the early detection of tumors. In addition, ultrasound scans of the pelvis open up the possibility to detect malignancies such as ovarian cancer. If imaging measures give an indication of a malignancy, tissue samples of suspicious tissue, isolated through minimally invasive methods, can be analyzed by immunohistochemical methods enabling the differentiation between benign and malignant cells. The determination of biomarkers in such tissue samples can be used for the evaluation of the degree of malignancy, as well as for the detection of cellular structures, which can serve targets for a therapy. The biomarker Ki-67 indicates the cell proliferation and thus gives important information regarding the growth behavior and the malignancy of the tumor [5]. The biomarker Her2/neu, on the other hand, does not only carry information about the malignancy of the tumor, but is also a direct object for a targeted cancer therapy with the drug Herceptin [6]. The definition of such points of attack for targeted therapies such as Herceptin allows a pharmacodiagnostic concept, in which the identification of biomarkers enable the identification of cancer patients eligible for targeted therapies. The detection of tumor-associated biomarkers in the blood is used for the detection of malignancies. CA-125, for example, is used as a tumor marker for detecting ovarian cancer [7]. The hopes placed in tumor/biomarkers have not, however, been fulfilled in many cases because they do not have the necessary sensitivity and specificity for early and reliable detection of malignancies. Thus, the very low specificity of the biomarker prostate specific antigen (PSA), which is used for the detection of prostate cancer, has led to many false positive results and concomitant considerable problems [8]. In contrast to the initial diagnosis of malignancies, biomarkers such as PSA, however, provide significantly better clinical information when used for the purpose of therapy monitoring and detection of return of cancer (recurrence). This allows the biomarker PSA, which is ultimately no tumor marker, but a tissue-specific marker, the detection of a return of prostate cancer, when previously the prostate was removed (prostatectomy). However, the sensitivity is not very high, because, not in all cases, the relapses and metastases in prostate cancer form the PSA, resulting in a lack of PSA increase misleadingly seen as a stable situation in the aftercare. Due to these false-negative results, cancer treatments are too late. A repeated increase in the concentration of a biomarker such as PSA in the blood, however, is usually a sure sign of a return of cancer. Since most of the tumor markers can be used only for particular tumor types, it is so far not possible to use tumor markers for a general detection of malignancies. Because of this, people are searching increasingly for biomarkers that allow general detection of tumors. Such a “pan-tumor” test would greatly simplify the diagnosis of all types of tumors and thus significantly improve the treatment as well as their monitoring. Therefore, earlier detection of tumors would increase the proportion of benign and also the less malignant tumors, which would increase the percentage of operable tumors. Because the surgical removal of tumors still represents a decisive and above all very successful therapy of tumor diseases, the establishment of diagnostics for the early detection of cancer is an important option to increase the success of this treatment by surgical tumor removal. So far, small benign tumors and small malignancies can be not detected with tumor markers, which are determined in the blood/serum, because they release relatively small amounts of a biomarker, which will also greatly be diluted in blood volume so that no significant increase in the concentration of biomarkers in the blood/serum is measurable.

## 2. Biomarker Detection with EDIM Technology

A novel method for the early detection of malignancies called epitope detection in monocytes (EDIM) detects biomarkers in special cells in the blood that carry tumor material within [1,2]. These cells are positive for cell surface markers CD14 and CD16, indicating that the monocytes represent activated monocytes (macrophages). The tumor material contained within these macrophages is not diluted by the blood volume and instead remains highly concentrated within the cells’ interior. This is an important factor determining the sensitivity of the EDIM technology. This method is based on the oldest and very effective purpose of the immune system: to identify and eliminate unwanted cell structures (phagocytose) by surrounding and digesting them. These unwanted cell structures may be foreign invaders, such as bacteria and parasites, or endogenous cells with abnormal growth and apoptosis, also known as tumor cells. After the macrophages phagocytosed the tumor cells or fragments of them, they digest the cells/cell fragments over a period of several days, during which time they leave the tumor and move through the vascular system to the bloodstream. Additionally, other tumor cells may be directly phagocytosed by the macrophages in the blood. The presence of tumor markers in blood cells that bear the surface markers CD14 and CD16 was first discovered by the presence of PSA in these cells [9]. These scavenger cells that contain tumor material in their cytoplasm (“stomach contents”) can be identified in whole blood samples by the fact that they bear the surface markers CD14 and CD16. These cell structures on the cells’ surface can be detected in blood samples very efficiently using dye-labelled antibodies and the subsequent detection of these dyes by means of laser beams through flow cytometry [1,2]. This makes it possible to detect macrophages in the blood, as well as to count and characterise these cells further.

The recognition and counting of macrophages in the blood alone is not sufficient for the early detection of malignancies, as there is no significant difference in the number of macrophages in healthy and cancer patients [10]. However, there is a significant difference between the number of macrophages containing tumor material in healthy subjects and in cancer patients [1,2]. To detect this subgroup of tumor-specific macrophages harboring tumor epitopes intracellularly, the macrophages in the blood must be treated so that holes form in their cell membranes. This allows the dye-coupled antibodies to reach the interior of the macrophage and thereby detect the cell structures of tumor cells in the intracellular compartment of the macrophages. When using this method, the number and size of holes in the cell membranes, as well as the length of time in which the holes are created, are very critical parameters. These parameters must be selected so that a sufficient number of dye-labelled antibodies pass into the macrophages, allowing for the identification of intra-cytoplasmic tumor material, but so that the excess dye-labelled antibodies from macrophage can escape. Thus, unspecific signals must be avoided. By mastering this complex and fragile test method, it has become possible to reliably detect tumor cell structures within macrophages [1,2,3,9,11,12,13,14]. This new technology for the tumor epitope detection in activated monocytes (macrophages) exploits the highly specific detection and elimination of tumor cells by the innate immune system. Therefore, sensitivity and specificity of the detection of tumor cells is strongly influenced by the sensitivity and specificity of the immune system and not only by the sensitivity and specificity of the tumor markers used. As a consequence, the sensitivity and specificity of the test is a combination of the sensitivity and specificity of the immune system and the sensitivity and specificity of the tumor markers used, enabling test sensitivities and specificities much better than sensitivities and specificities of the used tumor marker. Because every type of epitope present in macrophages as a result of phagocytosis can be used for the epitope detection in monocytes (EDIM), the EDIM technique represents a new technology platform for the detection of biomarkers including tumor markers. As part of the innate immune response, the activated monocytes (macrophages) reach every region of the human body to eliminate unwanted cells/cell structures. After that, macrophages come back to the blood and, as a consequence, the tumor material within can be analyzed by macrophages in the blood. Therefore, the basis of the EDIM technology is the already existing intrinsic liquid biopsy executed by the innate immune system. Combining this naturally occurring liquid biopsy with a flow cytometry based identification of macrophages and analysis of intracellularly present tumor epitopes, a new door is opened for the detection and characterization of cancer. A number of studies have shown sensitivity, and specificity in the detection of markers with EDIM technology is superior compared to free blood-floating biomarkers [11,12,13]. Biomarkers and EDIM technology can be used together in order to detect tumors earlier and with more specificity and specificity.

## 3. DNaseX: A Biomarker for Abnormal Apoptosis in Tumor Cells

A general test for malignancies would be possible only if there were specific biomarkers detectable in all forms of malignancies. The notion of finding such biomarkers seems unrealistic at first glance, since the development of malignant cells is a very complex, multistage process, starting from very different forms of healthy cells. As a result, the prevailing school of thought is that such biomarkers do not exist. If this is true, it would not be possible to establish a pan-test that can detect all forms of malignancies. However, new studies show that there are biomarkers that detect fundamental biophysical mechanisms that are significantly altered in all forms of malignancies [2]. Although the wide variety of disordered processes and signalling pathways among different tumor entities indicates a high complexity and heterogeneity, there are two fundamental processes that are altered similarly in all tumor cells. Malignant tumor cells (cancer cells) show a metabolic switch to an oxygen-independent energy release, but, first, all tumor cells have a disorder in the process of planned cell death (apoptosis). Only when this process is disrupted and, as a result, the cell no longer kills itself, a tumor cell can arise. Following this rationale, tumor cells consistently have biomarkers that demonstrate impaired apoptosis. Since the signalling cascades that lead to the induction of apoptosis are multifactorial, it is useful to look for a final step of apoptosis that leads to irreversible cell death. In this case, this final, irreversible step is the cutting of the chromosomal DNA into 300 base-pair pieces by enzymes called DNases. In the early 1990s, Harald zur Hausen, the then-head of the German Cancer Research Center in Heidelberg and later Nobel Laureate, initiated a molecular genome analysis pilot project to systematically identify all genes in the genomic region Xq28. This led to the discovery of a gene that encodes an enzyme to cut DNA into 300 base-pair fragments, and since this gene is located on the X chromosome, it was designated DNaseX [15]. The DNaseX protein had strong sequence similarities with the DNase I protein that has previously been discovered. Since the DNase I protein is required for the digestion of DNA from food, and since it is produced in large quantities in the pancreas, DNase I was initially considered a digestive enzyme, produced in the digestive tract. Only later was it discovered that the DNase I enzyme also plays an important role in the digestion of DNA in the blood—it digests the DNA from dead cells. If DNase I does not degrade the DNA released by dead cells into the blood, it can lead to the autoimmune disease Systemic Lupus Erythematosus (SLE). Further research showed that the inhibition of DNA degradation in unwanted cells (pre-malignant and malignant cells) happens and, thus, that apoptosis is inhibited in tumor cells [16,17]. For example, it could be shown that the expression of the DNase I enzyme consecutively decreases over the course of cervical cancer tumor genesis [17], whereas, in healthy cervical epithelium, a normal expression of the DNase I enzyme is still detectable. In cervical intraepithelial neoplasia (CIN), DNase I constantly moves to have the lowest level of activity in the invasive carcinoma. This emphasizes that the decreased presence of the DNase I enzyme is associated with the increase in the malignancy of CIN and cervical carcinoma. This suggests that the reduced presence of the DNase l enzyme contributes to a decreased rate of DNA degradation in cases of CIN and cervical cancer; thus, the cell does not perform apoptosis with sufficient strength, resulting in an increased survival of CIN and cervical carcinoma cells. The expression of the DNaseX enzyme in CIN and cervical carcinoma cells, in contrast, was increased compared to the healthy cervical epithelium cells (Coy, data not shown). At first glance, this paradoxical result arises from the fact that, although there is an increased expression of the DNaseX enzyme, which performs the final step of apoptosis, the tumor cell manages to inhibit the enzyme’s activity by forming inhibitors [16]. This prevents the cutting of tumor cells’ DNA, despite the presence of elevated concentrations of the DNaseX enzyme. This accumulation of blocked DNaseX was detected not only in CIN and cervical cancer cells, but also in all forms of pre-malignant and malignant cells investigated so far [2]. This is particularly well documented when the so-called Apo10 protein epitope of the DNaseX protein sequence is used for detection [2]. This block to the final step of apoptosis and the accumulation of DNaseX and the Apo10 protein epitope thus represent a general process in the development of pre-malignant and malignant tumor cells, which can now be diagnostically exploited.

## 4. Diagnostic Significance of the TKTL1 Gene

Professor zur Hausen’s systematic analysis of the genomic region Xq28 led to the discovery of another gene, *transketolase-like 1 (TKTL1)* [18], which forms the basis of an altered fundamental biophysical process in all tumor cells and represents the switch in energy released of oxidative phosphorylation towards fermentative energy release [19,20]. The *TKTL1* gene represents a significantly altered form of the transketolase (TKT) created in the course of vertebrate evolution [19]. The activation of the *TKTL1* gene in tumor cells leads to a fermentative, oxygen-independent metabolism, which is accompanied by an increased intake of glucose and an increased formation of lactic acid [21,22,23]. In 1924, Nobel Laureate Otto Warburg discovered this fermentative metabolism, which is not suppressed by oxygen, and called it “aerobic glycolysis” because it takes place despite the aerobic conditions and the so-called Pasteur effect (the suppression of fermentation by oxygen) [24]. Later, this particular form of fermentation was named the Warburg effect. Ever since the Warburg effect was reinterpreted as metabolism with protective function for healthy and malignant cells [19,20], rather than a form of cancer specific metabolism, it became a focus for many cancer researchers, so much so that the number of published studies on the Warburg effect increased exponentially. The fact that the Warburg effect is not present in all tumor cells, and that it is also present in many healthy cells, demonstrates an important function [19,20]. This fermentative metabolism, leading to the formation of lactic acid, even if oxygen is present, allows an acid-based degradation of the surrounding matrix. The result is a tissue remodelling and an invasive growth of cells. This represents an important process in the healing of wounds, as well as in the invasive growth of tumors. Since the transition from the non-invasive growth to the invasive growth of tumors represents a decisive factor in the increase in malignancy, which is associated with drastically shortened survival times, the Warburg effect and its fermentative metabolism have great clinical significance. The causal role of the activation of the *TKTL1* gene in tumor cells—which causes a switch to a fermentative metabolism and goes hand-in-hand with increased glucose intake, increased lactic acid production and invasive growth behavior—was recently proven again [23,25]. At the same time, the activation of the *TKTL1* gene in tumor cells leads to increased cell proliferation and the inactivation of apoptosis, which is normally triggered by the withdrawal of growth factors and hormones [26]. In addition, the activation of the *TKTL1* gene allows the avoidance of a radical formation, as by this fermentative metabolism ATP is generated through the substrate chain phosphorylation and, thereby, no radicals occur in this way of ATP production. Biophysically, the release of energy with the help of the TKTL1 enzyme differs substantially from the oxidative phosphorylation via mitochondria to the extent of the formation of free radicals and also, thus, to the extent of the cell-damaging side-effects. In addition, radicals are neutralized by metabolites of the fermentative metabolism, such as pyruvate, so that radicals, which are formed through exogenous factors such as sunlight or X-rays, can be neutralized. This plays a major role in the retina—in the suppression of radicals formed by sunlight, for example. While using mitochondrial oxidative phosphorylation makes possible a very effective release of energy, because glucose is completely broken down into water and carbon dioxide, the fermentative release of energy is less effective at first glance because the glucose is partially broken down and the energy-rich molecule lactic acid is emitted outside the cell. However, though this lactic acid is not excreted from the body, it continues to be used, and therefore no energy is lost by whole organisms. The fermentation is thus not an energy loss, but an effective pathway, so long as there is enough glucose present and the lactic acid can be released from the cell. In fact, it is the only way for the energy to be released when no oxygen is available for oxidative phosphorylation. It is also the only way for an energy release when the formation of free radicals must be avoided. Since the formation of radicals is a process that damages both healthy and malignant cells, it is important to develop strategies to prevent their formation. For example, the highest activity of fermentative energy release, or aerobic glycolysis, and the highest concentration of TKTL1 exist in a man’s testicles [19]. The reason for this fermentation even in the presence of oxygen is the production of DNA in sperm cells, which is not damaged by free radicals. If the DNA in sperm is exposed to the free radicals, which are generated through oxidative energy release, then it would significantly increase the number of mutations in sperm and thereby the number of disabled children, which would decline the fertility of man. Therefore, it is not surprising that the TKTL1 protein is one of the most important markers of fertility in men [27]. The fermentative metabolism also plays a crucial role in stem cells because mutations in stem cells have particularly negative effects, as they are used to regenerate many cells and tissues. Both healthy stem cells and cancer stem cells use the fermentative metabolism to prevent the formation of free radicals. The protective function of the fermentative metabolism for the survival of the organism as a whole has also been demonstrated [28]. Werner syndrome (WS), for example, causes rapid aging and premature death. An increased production of radicals and increased DNA damage can be found in WS patients’ cells. A genetic mutation can lead to the shutdown of the fermentative metabolism and the expression of the TKTL1 protein in cells, resulting in an increased production of radicals and DNA damage [28]. As a result, the life expectancy shortens dramatically. This shows how important the fermentative metabolism is for slow aging and having a long life. Ultimately, this fermentative pathway represents a strategy for preventing radical-associated cell damage and for allowing for the longer survival of individual cells and the entire organism [28,29].

## 5. The Second Way of Lactic Acid Fermentation in Mammals: A Great Moment during Evolution of Vertebrates

Remarkably, aerobic glycolysis, the fermentation even in the presence of oxygen (Warburg effect) and its enzymatic basis are not described in biochemistry or medicine textbooks. In textbooks, lactic acid production in humans is described only as the result of a lack of oxygen, as it occurs in skeletal muscle if there is not enough oxygen for combustion. This oxidative phosphorylation in the mitochondria is switched off because there is not enough oxygen for the oxidation of hydrogen. Then the vital energetic connection of ATP can be made only with the help of fermentative metabolism. A glucose molecule with six carbon atoms is broken into two halves with three carbon atoms each and is used as an acceptor for hydrogen, with the help of the Embden–Meyerhof pathway, to form two lactic acid molecules. Thus, it is possible to break down glucose without oxygen, thereby creating ATP. The price for this, however, is the formation of lactic acid, which can be formed only up to a certain concentration before the pH of the tissue, the blood or of the entire body becomes too acidic.

In the short term, lactic acid production as a result of the lack of oxygen is an excellent survival mechanism—for maintaining skeletal muscle activity when the skeletal muscles lack a sufficient oxygen supply, for example. This allowed our hunter–gatherer ancestors to practice fight or flight, even if their skeletal muscles were no longer adequately supplied with oxygen. After winning the battle or escaping, the hunter–gatherers could rest, and the lactic acid formed was thereby broken down through oxidation, using the then-sufficient oxygen present. Unlike the skeletal muscles, the heart muscle does not use this emergency fermentation because the heart muscle is much better supplied with oxygen, due to its proximity to the lungs. The heart also contributes to fight-or-flight actions through its oxidative degradation of the lactic acid formed in the skeletal muscles, so that the skeletal muscles can form lactic acid more and for longer. The heart muscle contributes significantly to a lactic acid organ interexchange between skeletal muscles and heart muscle, which increases and extends the performance of the skeletal muscles. This also explains why the heart muscle prefers the use of lactic acid during oxidative energy release, as it preserves the blood glucose and makes it available to the skeletal muscles. When sufficient oxygen is present in skeletal muscles, it begins to downshift using an oxidative metabolism, so no more lactic acid is formed. This effect of the suppression of fermentation by oxygen is named after the discoverer of this principle—the Pasteur effect. In 1924 in the study of the metabolism of carcinoma tissues, Nobel Laureate Otto Heinrich Warburg noted that the suppressive effect of oxygen on this special fermentation metabolism does not exist [24]. In contrast, the skeletal muscle of frogs he examined in these experiments showed the “Pasteur effect” because the fermentation of glucose to lactic acid was stopped immediately by the effect of oxygen. Carcinomas performed a fermentation leading to lactic acid even when sufficient oxygen for combustion was present. Warburg called this fermentation despite the presence of oxygen “aerobic glycolysis” [24]. He saw it as the root cause of cancer. Warburg had also examined retinas and testicles of rats in his experiments for fermentation in carcinomas in addition to the skeletal muscle of frogs and found that these healthy tissues also have a formation of lactic acid in the presence of oxygen (aerobic glycolysis). This makes it clear that this form of fermentation is not limited to carcinomas but is also present in healthy tissues. Warburg could not tell the reasons for the use of aerobic glycolysis in healthy tissues such as the retina and testicles but postulated, anyway, that the aerobic glycolysis is the main cause of cancer. This unresolved contradiction increased the rejection of many cancer researchers towards Otto Heinrich Warburg’s theory of the metabolic cause of cancer. These resistors have been further strengthened, discovered the role of cancer-causing viruses such as Rous sarcoma virus in the following decades, and DNA mutations have been identified that have played a significant role in the development of cancer. Warburg’s theory that the anaerobic metabolism plays a crucial role for cancer has been confirmed by Nobel laureate Albert Szent-Györgyi. He divided life into a first anaerobic period, the so-called alpha period (alpha state), followed by a second aerobic period (beta period), where oxygen is the universal electron acceptor [30]. Albert Szent-Györgyi postulated: “During the subsequent aerobic beta period, more highly differentiated life forms could develop because of occurrence of O_2_, a strong electron acceptor leading to a greater degree of desaturation. When dividing, however, beta-type cells return partially to the proliferative alpha state” [30]. Therefore, Albert Szent-Györgyi realized that dividing cells including cancer cells perform a metabolic switch to an oxygen-independent energy metabolism—the anaerobic alpha state. The discovery of the *TKTL1* gene as the basis of a novel mammalian specific oxygen-independent energy metabolism similar to the heterofermentative lactic acid bacteria enabled the reinterpretation of Warburg’s and Szent-Györgyi’s findings that the metabolic switch to an oxygen-independent energy metabolism is a protective metabolism in proliferative cells—in cancer cells and in normal cells [19,20]. Furthermore, the enzymatic reaction of TKTL1 explains the difference between the tentatively proposed reactions of the nonoxidative branch of the pentose phosphate pathway (PPP) presented in textbooks and the actual observed results gained by ^14^C isotope labeling. Although the PPP represents a basic biochemical pathway, the proposed reactions of the nonoxidative branch of the PPP presented in textbooks are still controversial because the degree of ^14^C isotope labeling and its distribution in carbon atoms of fructose-6-phosphate differed from that predicted by reaction sequences. Horecker and co-workers, the authors who described the nonoxidative branch reactions of the PPP detected this disquieting difference between their tentatively proposed reaction scheme and their experimental results: “However, from the results with labeled pentose phosphate, it is apparent that the transketolase–transaldolase sequence of reactions, as depicted in the introduction, is by itself insufficient to account for hexose monophosphate formation, since the distribution of isotope in the product differs substantially from that predicted by these reactions” [31]. Surprisingly, the notable difference between the tentatively proposed reaction scheme and the experimental results received uncritical approval from PPP reviewers and textbook authors from that time.

**Figure 1:**
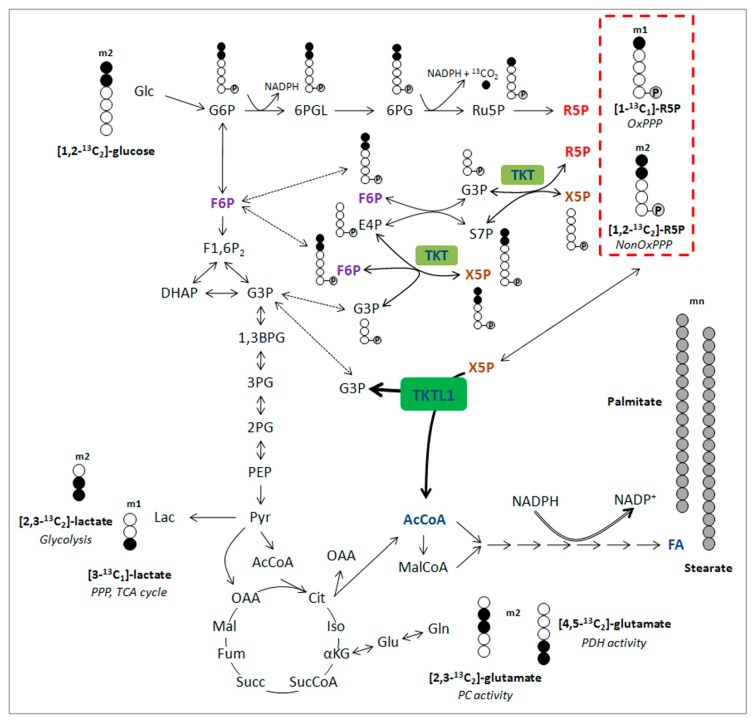
Overview of isotopomer formation through carbon distribution of [1,2-^13^C_2_]-glucose. Distribution of carbon atoms from [1,2-^13^C_2_]-glucose results in the presence of different kinds of isotopomers from lactate, glutamate and ribose-5-phosphate and reflect the involvement of specific pathways (italic). Ribose-5-phosphate incorporating 3, 4 or 5 ^13^C atoms (m3, m4 and m5) is generated through recirculation of labeled molecules in the pentose phosphate pathway PPP. Carbons are represented by circles. Filled circles represent carbons that have incorporated label form [1,2-^13^C_2_]-glucose, open circles represent unlabeled carbons. Circles containing P represent phosphate groups. Abbreviations: m1, m2, isotopologues; 1,3BPG, 1,3-bisphosphoglycerate; 2PG, 2-phosphoglycerate; 3PG, 3-phosphoglycerate; 6PG, 6-phosphogluconate; 6PGL, 6-phosphogluconolactone; AcCoA, acetyl-CoA; Cit, citrate; DHAP, dihydroxyacetone phosphate; E4P, erythrose-4-phosphate; FA, fatty acids; F1,6P2, fructose-1,6-bisphosphate; F6P, fructose-6-phosphate; Fum, fumarate; G3P, glyceraldehyde-3-phosphate; Glc, glucose; G6P, glucose-6-phosphate; Iso, isocitrate; Lac, lactate; Mal, malate; MalCoA, malonyl-CoA; NonOx, nonoxidative; OAA, oxaloacetate; PC, pyruvatecarboxylase; PDH, pyruvatedehydrogenase; PEP, phosphoenolpyruvate; Pyr, pyruvate; R5P, ribose-5-phosphate; Ru5P, ribulose-5-phosphate; S7P, sedoheptulose-7-phosphate; Succ, succinate; SucCoA, succinyl- CoA; TCA, tricarboxylic acid; TKT, Transketolase; TKTL1, Transketolase-like 1; X5P, xylulose-5-phosphate; αKG, α-ketoglutarate. Figure published in [25].

The *TKTL1* gene represents a form of a transketolase that was created in the course of vertebrate evolution leading to a mammalian specific gene [18]; specifically, it has a deleted exon encoding conserved amino acid residues important for the enzymatic reaction modus of transketolase enzymes. Through the deletion of this exon and the concomitant removal of the conserved amino acid residues, the typical transketolases’ two-substrate reaction shifted to a preferred one-substrate reaction. As a result of this modified enzyme reaction, lactic acid and acetyl-CoA are formed and acetyl-CoA is used directly for fat synthesis [25] (Figure 1). Compared to the generation of acetyl-CoA by pyruvatedehydrogenase as the downstream enzyme of the Embden–Meyerhof pathway, the formation of acetyl-CoA by TKTL1 is superior because no carbon atom is lost by the generation of carbon dioxide. This means that an atom of carbon per pyruvate is lost—about a third of the carbon atoms, if acetyl-CoA is produced by the pyruvatedehydrogenase enzymatic reaction modus. If we take glucose as the starting substrate, this means that two of six carbon atoms are lost when a cell produces acetyl-CoA from glucose. This situation exists in cells that want to reproduce. Acetyl-CoA is one the most important metabolites for the synthesis of cellular components, such as lipids and fat. The conversion of sugar into fat via the Embden–Meyerhof pathway is extremely inefficient because one-third of the sugar is lost by the formation of carbon dioxide. The formation of acetyl-CoA from sugar with the help of the TKTL1 enzyme, however, is extremely efficient because all carbon atoms of glucose are converted into fat, and no valuable raw materials are lost. In addition to the formation of a high-energy connection in the form of acetyl-CoA, the TKTL1 enzyme allows for the formation of pyruvate, as the hydrogen can be transferred and lactic acid is formed. This allows the regeneration of NADH + H^+^ and ATP production via fermentation. The ability to break down sugars with five carbon atoms into acetyl-CoA and lactic acid was formed very early on in evolution and is still used today by hetero-fermentative lactic acid bacteria to break down glucose. The key enzyme for this reaction is the phosphoketolase enzyme, which belongs to the family of thiamine-dependent enzymes. That the TKTL1 enzyme is capable of acetyl-CoA incorporation in fat [25] represents a milestone in evolution because a mutation was able to change a transketolase enzyme reaction so that a new reaction resulted in lactic acid and acetyl-CoA generation. Bacteria several billion years ago already established a so-called phosphoketolase enzyme forming lactic acid and acetyl-CoA generation from sugars with five carbon atoms. This means that in mammals next to the Embden–Meyerhof pathway, a phosphoketolase-like enzymatic reaction (TKTL1) also allows a degradation of sugar into acetyl-CoA and lactic acid. Therefore, in view of evolution, the TKTL1 enzymatic reaction represents a re-invention of the phosphoketolase reaction, allowing mammals a more efficient way of acetyl-CoA synthesis and an energy release free of radicals even in the presence of oxygen. In contrast to the lactic acid generation by the Embden–Meyerhof pathway and due to the benefit of the TKTL1 enzymatic reaction, it is performed in the absence and in the presence of oxygen. This type of fermentation is performed in the presence of oxygen, as damage caused by oxygen or high-energy rays can be suppressed. In the retina, light-induced damage and blindness are counteracted since no other radicals are formed in the mitochondria and since the other metabolites involved in this fermentation metabolism, such as the pyruvate, have a radical neutralizing effect. In the testicles, the blocking and suppression of radical formation are also made possible by the TKTL1 fermentative metabolism, which protects the DNA in sperm from DNA mutations, which are triggered by radicals. Due to the high glucose flux of the TKTL1 enzymatic reaction, testicles are also a good example for demonstrating high glucose intake (“hot eggs”) in ^18^FDG-PET scans. With an increase in the size of a tumor, there is a corresponding increase in the radical load and the oxygen deficiency (hypoxia); the activation of TKTL1 and the fermentative metabolism in tumor cells represents a selective survival advantage, as the radical production is reduced or neutralized and tumor cells can grow without oxygen. Therefore, the RedOx homeostasis is positively influenced by the fermentative TKTL1 metabolism and the oxidative part of the pentose phosphate pathway. Sufficient reduction equivalents such as NADPH + H^+^ and glutathione are formed, which carry out the reduction of cytochrome c. This, in turn, has the consequence that the initiation of apoptosis is suppressed. The fermentative metabolism not only neutralizes radicals, but radical formation is furthermore avoided, and due to the reduction of cytochrome c, the induction of apoptosis is inhibited. Thus, in nerve cells, as well as in cancer cells, the triggering of apoptosis via the cytochrome c in the mitochondria is suppressed, and so cell death is avoided [32]. For neurons, this represents an important survival factor. Studies have shown that nerve cells do not die when beta amyloid plaques are present. The condition, however, was that aerobic fermentation (Warburg effect) had to occur [33]. Nerve cells without this protective type of fermentation did not survive the formation of beta amyloid plaques. Therefore, the fermentative metabolic pathway in the presence of oxygen provides the crucial protective factor for cells and, consequently, for the whole organism. Healthy cells can thus be protected from cell damage by free radicals and from premature death. This protection program, developed in the course of vertebrate evolution, is specific to mammals and is one of the most important explanations resulting from the study of evolution. As a result of this discovery, mammals were able to develop high and long-lasting functionality in the nervous system and the brain. The function and dysregulation of TKTL1 and DNaseX/Apo10 in healthy and cancer cells is summarized in Table 1.

## 6. The Clinical Significance of Activating the *TKTL1* Gene

**Figure 2:**
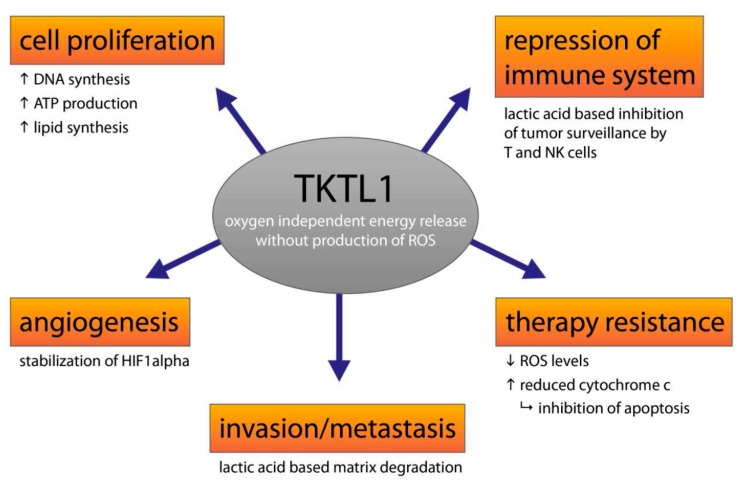
Role of TKTL1 in cancer cells. Contribution of TKTL1 and its metabolites to important hallmarks of cancer leading to increased malignity, survival, immune escape, therapy resistance and distribution of cancer cells in the body. “arrow up” indicates increase, arrow down indicates decrease.

Today, the TKTL1 gene is important for Homo sapiens as it is a key factor in the evolution human cognitive performance [34]. Unfortunately, the activation of this protection program in unwanted tumor cells also has protective effects against the prevention of radicals (ROS), cell damage and the suppression of the cell death trigger. In tumors, the *TKTL1* gene leads to a resistance to radical and apoptosis inducing therapies, so cancer patients are unable to benefit from neo-adjuvant chemotherapy and radiation [35]. The TKTL1 activity, e.g., also correlates with the proliferation and progression of cervical cancer [36,37] and lymph node metastasis in thyroid carcinoma [38]. Furthermore, the activation of the TKTL1 gene in tumor cells results in an increased glucose intake and lactic acid formation, invasive growth, the formation of distant metastases and an increased relapse rate [20,23,35,39,40,41,42]. The malignancy of TKTL1-positive tumors is also increased because the lactic acid produced leads to an acid-based inhibition of immune cells and tumor cells that cannot be eliminated effectively by immune cells, like the killer cells [43,44]. Additionally, because apoptosis-triggering is inhibited by the withdrawal of growth factors, TKTL1-positive tumor cells are more resistant against anti-growth strategies e.g., anti-hormone therapies [26]. These individual effects lead to a significant increase in the malignancy of tumors with TKTL1-expression (Figure 2) and to a drastically shortened survival time for patients [20,23,35,39,40,41,42]. At the same time, the sensitivity against radical and apoptosis-triggering therapies, which makes the tumor cells more resistant to radiation and chemotherapies [35], is reduced through the activation of the *TKTL1* gene. Furthermore, the fermentation metabolism also leads to a resistance to targeted therapies, such as sorafenib [45], imatinib [46] and cetuximab [47]. In the case of sorafenib, the activation of the fermentative metabolism leads to a suppression of free radicals and, thus, to resistance to sorafenib. If the fermentative metabolism is inhibited by the drug oxythiamine, this results in the efficacy of sorafenib [45]. Imatinib also could be made effective once again through the inhibition of the fermentative metabolism by oxythiamine [46]. Furthermore, oxythiamine was successfully used to inhibit the growth of ovarian carcinoma cells in patients with chemo-resistant ovarian cancer [48]. In addition to the small compound based inhibition of fermentative metabolism, knockdown of TKTL1 additively complements cisplatin-induced cytotoxicity by inhibiting the levels of NADPH and ribose-5-phosphate, indicating that TKTL1 may be a promising target to improve the therapeutic effect combining with cisplatin for cancer patients [49]. This indicates that it is possible to inhibit the fermentative metabolism, to inhibit the growth of chemo-resistant cancer cells and to increase the effectiveness of therapies by combining them to combat drug resistance.

## 7. Benfo-Oxythiamine: The Vitamin Antagonist Oxythiamine in a New Dress

Studies on oxythiamine indicate that analogues of vitamins can be used to successfully treat cancer. With the establishment of methotrexate as folic acid (vitamin B9), it was possible to inhibit the dihydrofolic reductase, a key enzyme in the biosynthesis of folic acid. Methotrexate succeeds in blocking the folate-dependent production of RNA and DNA so that important elements for the growth of cancer cells are absent. In the case of vitamin B1 (thiamine) antagonist oxythiamine, it is possible to inhibit thiamine-dependent enzymes, such as the transketolases, as well as the pyruvatedehydrogenase and the alpha-ketoglutaratedehydrogenase. The sugar metabolism can be inhibited extremely efficiently in several places with oxythiamine, as the thiamine-dependent enzyme reactions of transketolases (TKT and TKTL1), the pyruvatedehydrogenase and the alpha-ketoglutaratedehydrogenase control the Embden–Meyerhof pathway, the pentose phosphate pathway and the citric acid cycle (Krebs cycle)—or, in other words, the overall metabolism of sugar. This simultaneously results in an increased formation of radicals, oxidation of cytochrome c and inhibition of DNA repair. Due to these cancer cells becoming sensitive to radical and apoptosis-triggering therapies, such an effect has been proven in studies. Oxythiamine not only leads to the inhibition of DNA and RNA synthesis, but it also increases the formation of radicals (reactive oxygen species—ROS) and thus massively alters RedOx homeostasis. Both effects synergistically inhibit the growth of cancer cells and increase the efficacy of cancer therapies. Since the transketolase enzyme reaction is a crucial element in the formation of lactic acid, this can be addressed by inhibiting this enzyme reaction and by suppressing lactic acid-mediated matrix degradation, leading to the reduced invasiveness and metastasis of cancer cells. Since there are currently no medications that can successfully inhibit the invasiveness and metastasis of cancer cells, this therapeutic effect is of great importance for future cancer treatments. Having the option to inhibit the invasion and metastasis of cancer would amount to a paradigm shift in the field of oncology. Since the lactic acid formation of cancer cells also has a suppressive effect on the elimination of cancer cells by the immune system [43,44], the inhibition of the glucose metabolism also opens up possibilities for developing more effective immune therapies.

The abovementioned effects of oxythiamine are part of a large body of research indicating that the drug is a promising approach to drug therapy for cancer patients. Despite these sensational data on its effects, it is highly unlikely that it will be available as a cancer treatment in the future. Oxythiamine and its effects have been known for many decades, making its application in cancer no longer new and no longer patentable. Pharmaceutical companies have therefore dropped oxythiamine as an active ingredient in the clinical development of new drugs. Without patent protection for pharmaceutical companies, it is not possible for them to recover their substantial financial investments in clinical drug development since competitors can bring in a non-patented drug quickly and at a low cost, and thereby compete with them on the market. Therefore, it is very likely that oxythiamine will join the family of well-known anti-cancer drugs that are clinically effective but unapproved and, therefore, non-applicable. One way out of this dilemma could be the potential establishment of benfo-oxythiamine (BOT), a previously unknown thiamine analogue that releases oxythiamine in the human body, as a completely new and patentable drug. This opens up possibilities for clinical development with a patented active ingredient that is ultimately a prodrug of oxythiamine. Since benfo-oxythiamine can now be produced in larger quantities under good manufacturing practice (GMP) conditions, the clinical evaluation is possible.

## 8. EDIM-TKTL1/Apo10 Blood Test: An Innate Immune System Based Liquid Biopsy for the Detection of Cancer Patients Eligible for a Targeted Treatment

Due to the increased malignancy and the enhanced therapy resistance of tumors with TKTL1 fermentation metabolism, such cancer patients have a significantly worse survival probability. The detection of TKTL1 in tumor material offers the possibility to identify this subset of patients and to be monitored by close-knit diagnostics and to accurately apply early and targeted therapies. At the same time, the detection of the TKTL1 fermentation metabolism in tumors opens up the identification of tumors in which a sensitization to cancer therapies is necessary. In the future, a pharmacological inhibition of glucose metabolism can be made by means of the vitamin antagonists benfo-oxythiamine or oxythiamine prior to chemotherapy or radiation therapy, so that the formation of radicals and apoptosis is increased, facilitating the death of cancer cells. Through this pharmacological inhibition of glucose metabolism, a new therapeutic option is available to allow a significant extension of survival time and cures of cancer patients with previously poor prognosis. In addition, the EDIM blood test allows the detection of TKTL1 as well as of all other drug targets, enabling an individualized cancer therapy addressing actual existing targets in tumors. Therefore, the EDIM blood test will be an important and necessary part of targeted therapies.

## 9. EDIM-TKTL1/Apo10 Blood Test for the Detection of Patients Eligible for ^18^FDG-PET Imaging of Tumors

The metabolic characterization of tumors is possible with this blood test, since elevated TKTL1 levels in macrophages correlated with an increased glucose uptake by the ^18^fluor-desoxyglucose (FDG) PET-imaging, and so tumor patients eligible for ^18^FDG-PET imaging can be identified by the EDIM-TKTL1 blood test [1]. This pre-selection of tumor patients enables the cost-effective application of ^18^FDG-PET, since the number of positive examinations is strongly increased. Furthermore, the EDIM-TKTL1 blood test identifies cancer patients with an enhanced glucose metabolism concomitant with the option for a detailed visualization and localization of tumors in the whole body, which is the basis for an improved therapy. Overall, the EDIM technology allows the identification of biomarkers that can be used for visualization and targeted therapies. This can be performed by the detection of targets like receptors, e.g., gastrin-releasing peptide receptors followed by a targeted treatment with radiolabeled agonists or antagonists thereof.

## 10. EDIM-TKTL1/Apo10 Blood Test for the Monitoring of Cancer Therapies

Since the EDIM-TKTL1/Apo10 blood test is based on the activity of the innate immune system, cancer treatments influencing the immune system and the activity of macrophages will affect the test results. Since most cytotoxic cancer treatments do have such an effect on the activity of macrophages, cytotoxic and other cancer treatments influencing the immune system cannot be monitored by the EDIM technology.

## 11. EDIM-TKTL1/Apo10 Blood Test for the Monitoring of Surgical Removal of Tumors

With the help of a EDIM-TKTL1/Apo10 blood test, the surgical removal of the tumor can be monitored [2,3] (Table 2). Comparison of pre- and postoperative scores of the EDIM blood tests indicates if the tumor has been successfully removed by surgery. This is an important application of the EDIM-TKTL1/Apo10 test because cancer patients can be detected who need further surgical removal of remaining tumor material or another form of therapy.

## 12. EDIM-TKTL1/Apo10 Blood Test for the Early Detection of Cancer Recurrence

With the EDIM-TKTL1/Apo10 blood test, the early detection of cancer recurrence was possible [4]. In this case, the EDIM blood test allowed a much earlier detection of recurrence than application of tumor marker detection in serum. Future studies have to be performed to prove if this is in general the case.

## 13. EDIM-TKTL1/Apo10 Blood Test for the Early Detection of Cancer

**Figure 3:**
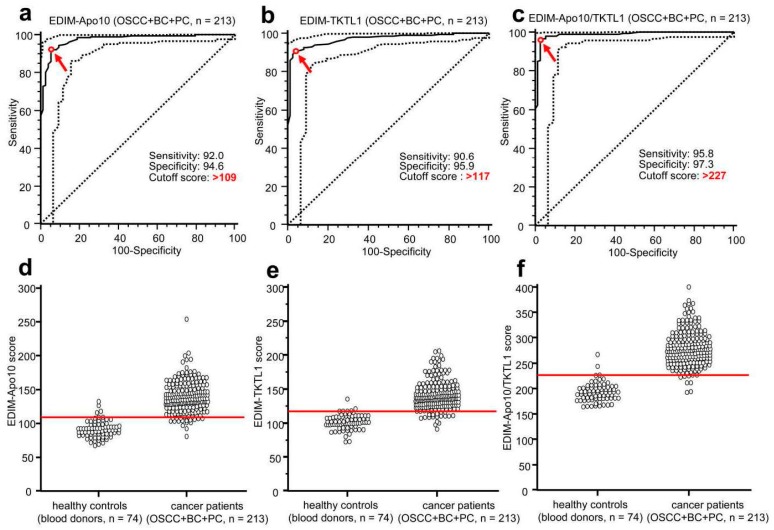
Receiver Operating Characteristics (ROC) analysis of epitope detection in monocytes (EDIM)-Apo10, EDIM-TKTL1, and combined EDIM Apo10/TKTL1 score in all cancer samples (OSCC, breast and prostate cancer, *n* = 213) compared with healthy individuals (*n* = 74). The true positive rates (sensitivity) are plotted in functions of the false positive rate (100-specificity) for measurement of the cut-off point: ROC analysis for the diagnosis of all cancer samples/entities (OSCC, breast and prostate cancer, **a**–**c**) shows calculated cut-off value with highest diagnostic accuracy (arrows) of EDIM-Apo10 (**a**), EDIM-TKTL1 (**b**), and combined EDIM Apo10/TKTL1 (**c**) score (**a**, EDIM-Apo10 score >109: sensitivity 92.0%, 95% confidence interval (CI) 87.5–95.3%, specificity 94.6%, 95% CI 86.7–98.5%; **b**, EDIM-TKTL1 score >117: sensitivity 90.6%, 95% CI 85.9–94.2%, specificity 95.9%, 95% CI 88.6–99.2%; **c**, combined EDIM-Apo10 plus EDIM-TKTL1 score >227: sensitivity 95.8%, 95% CI 92.1–98.0%, specificity 97.3%, 95% CI 90.6–99.7%). Dotted lines show 95% CI. OSCC, oral squamous cell carcinoma; BC, breast cancer; PC, prostate cancer. In the interactive dot diagrams (part of ROC curve analysis, **d**–**f**), the data of healthy controls and cancer group are displayed as dots on two vertical axes. The horizontal line indicates the cut-off points with the best separation/highest accuracy (minimal false negative and false positive results) between healthy controls and cancer group. The corresponding test characteristics sensitivity and specificity are shown above. Figure published in [2, additional file 13].

Evidence of biomarkers DNaseX/Apo10 and TKTL1 in macrophages by means of a blood test allowed the early detection of all cancer types analyzed so far [1,2,4]. Whereas the sensitivity and specificity of EDIM-Apo10 (92.0 and 94.6, respectively) and EDIM-TKTL1 (90.6 and 95.9, respectively) was very good, the combined score of EDIM-Apo10 and EDIM-TKTL1 was superior, leading to the extraordinary sensitivity of 95.8% and specificity of 97.3%, with the breast, oral cavity, and prostate cancers being detected [2] (Figure 3). This underlines the diagnostic potential of this new blood test procedure and demonstrates that if the results of the two biomarkers indicating two different fundamental biophysical mechanisms are being used together for the detection of malignancies, the specificity and the sensitivity are significantly higher. The specificity of the test results might be even higher because blood donors have been used as negative control, but these blood donors do not represent true negative controls because it is unknown if they bear a tumor. Thus far, the results of the present studies confirm that the EDIM-TKTL1/Apo10 blood test is a promising diagnostic method for the early detection of all types of malignancies.

## 14. Future Directions and Limitations

The EDIM-TKTL1/Apo10 blood test could be a “pan-tumor” blood test, which greatly simplifies the diagnosis of all types of tumors and thus significantly improves the treatment success. Therefore, earlier detection of tumors would increase the proportion of benign and also the less malignant tumors, which would increase the percentage of operable and non-metastasized, curable tumors. Because the surgical removal of tumors still represents a decisive and above all very successful therapy of tumor diseases, the establishment of diagnostics for the early detection of cancer is an important option to increase the success of surgical tumor removal and/or of current cancer treatments like radio-, chemo- and targeted therapies. Thus far, small benign tumors and small malignancies can be not detected with tumor markers, which are determined in the blood/serum, because they release relatively small amounts of a biomarker, which will also be greatly diluted in blood volume, so that no significant increase in the concentration of biomarkers in the blood/serum is measurable. The EDIM technology with the intrinsic help of the innate immune system solves this problem. Despite this, a “pan-tumor” blood test will be a challenge because the early detection of (small) tumors will significantly increase the number of detected tumors and concomitantly will raise the number of tumor patients. Although individuals with such small tumors do have a very good chance for being cured, the application of such a pan-cancer test should be connected to an education about the benefit and increased treatment success caused by the early detection of tumors.

## Figures and Tables

**Table 1:** The biological role of DNaseX/Apo10 and TKTL1 protein in healthy cells and the impact of activation/inhibition leading to the different diseases.

Biomarker	Healthy Cell	Function	Inhibition/Absence > Disease	Activation > Disease
DNaseX/Apo10	endonuclease activity leading to 300 bp DNA fragments	executing final step of apoptosis leading to the elimination of unwanted cells/tumor cells	abnormal cell proliferation and inhibition of apoptosis > arise of tumor cells	
TKTL1	energy release from carbohydrates, glucogenic amino acids and glycerin without generation of ROS	avoiding production of ROS and protection from ROS induced cell damages in particular in retina, testis and stem cells	high levels of ROS leading to cell damages, enhanced aging and premature cell death leading to (a) Werner-Syndrome (b) male infertility (c) premature neuron death/neurodegeneration (e.g., Alzheimer)	activation in tumor cells leads to prevention and suppression of ROS, reduction of cytochrome c concomitant with increased malignity of tumor cells, inhibition of apoptosis and increased therapy resistance towards radiotherapy, many chemotherapies (e.g., platin derivatives) and targeted therapies like imatinib, sorafenib.
	conversion/degradation of carbohydrates, glucogenic amino acids and glycerin to acetyl-CoA without loss of carbon atoms	generation of acetyl-CoA as the most important metabolite for synthesis of lipids for cell proliferation. TKTL1 dependent generation of acetyl-CoA is used for anabolic conditions, whereas pyruvatedehydrogenase dependent generation of acetyl-CoA is used for catabolic conditions and energy release by citric acid (Krebs) cycle	inhibition of TKTL1 by thiamine deficiency increase/induce chronic diabetes complications. Inhibition of TKTL1/TKT by oxythiamine created by certain cooking conditions leads to end-stage renal disease.	
	conversion/degradation of carbohydrates, glucogenic amino acids and glycerin to ribose and desoxyribose for DNA/mRNA synthesis and increased generation of NADPH by the oxidative part of the pentose phosphate pathway	generation of DNA and mRNA. Control of RedOx homeostasis by NADPH and glutathione.	enhanced DNA damage in healthy cells lead to premature aging	enhanced repair of DNA damages in tumor cells leads to therapy resistance towards radio- and many chemotherapies e.g., platin derivatives
	oxygen independent energy release and production of lactic acid even in the presence of oxygen and stabilization of HIF1α	cell survival under hypoxic conditions represents a survival mechanism for cells with no more access to blood oxygen caused by ischemia or infarct	absence of lactic acid based matrix degradation and invasive growth lead to inhibition of wound healing e.g., in diabetes patients	activation in tumor cells leads to a lactic acid based matrix degradation and concomitant to invasive growth and metastasis as well as resistance towards anti-angiogenic treatment e.g., avastin and erbitux
				activation in tumor cells leads lactic acid based inhibition of T and NK cells = protection from immune system attack
	confers cell survival and resistance to growth stimulus withdrawal e.g., hormone ablation	allows survival as single cell migrating in the body		resistance towards hormone ablation therapy e.g., androgen ablation therapy

**Table 2:** Pre- and post-operative epitope detection in monocytes (EDIM)-Apo10 and TKTL1 scores in patients with (**a**) oral squamous cell carcinoma (**b**) breast cancer (**c**) prostate cancer. Table published in [2].

(**a**) Pre- and postoperative epitope detection in monocytes (EDIM)-Apo10 and TKTL1 scores in patients with oral squamous cell carcinoma (*n* = 3).					
**Characteristics**		**Preoperative**		**Postoperative**	
Patients	Total *n* = 3	Apo10 score	TKTL1 score	Apo10 score	TKTL1 score
Patient 1		143	134	102	111
Patient 2		119	146	99	102
Patient 3		124	121	100	93
(**b**) Pre- and postoperative epitope detection in monocytes (EDIM)-Apo10 and TKTL1 scores in patients with breast cancer (*n* = 3).					
**Characteristics**		**Preoperative**		**Postoperative**	
Patients	Total *n* = 3	Apo10 score	TKTL1 score	Apo10 score	TKTL1 score
Patient 1		161	129	81	100
Patient 2		126	155	96	77
Patient 3		133	132	98	89
(**c**) Pre- and postoperative epitope detection in monocytes (EDIM)-Apo10 and TKTL1 scores in patients with prostate cancer (*n* = 6).					
**Characteristics**		**Preoperative**		**Postoperative**	
Patients	Total *n* = 6	Apo10 score	TKTL1 score	Apo10 score	TKTL1 score
Patient 1		155	149	98	99
Patient 2		144	166	93	106
Patient 3		153	165	93	101
Patient 4		162	143	105	104
Patient 5		139	149	102	88
Patient 6		158	144	95	95
